# *In vitro* activity of immunosuppressive drugs against *Plasmodium falciparum*

**DOI:** 10.1186/1475-2875-13-476

**Published:** 2014-12-04

**Authors:** Luzia Veletzky, Khalid Rehman, Tilman Lingscheid, Wolfgang Poeppl, Felix Loetsch, Heinz Burgmann, Michael Ramharter

**Affiliations:** Department of Medicine I, Division of Infectious Diseases and Tropical Medicine, Medical University of Vienna, Währinger Gürtel 18-20, 1090 Vienna, Austria; Department of Infectious Diseases and Respiratory Medicine, Charité Universitätsmedizin, Berlin, Germany; Institut für Tropenmedizin, Universität Tübingen, Tübingen, Germany

**Keywords:** *Plasmodium falciparum*, Immunosuppressives, Mycophenolic acid, Rapamycin, Everolimus

## Abstract

**Background:**

Solid organ transplant recipients are particularly vulnerable for infectious diseases due to prolonged immunosuppressive treatment. Residents of endemic regions and travellers may be exposed to malaria and may, therefore, require prolonged antimalarial chemoprophylaxis. The hypothesis of this study was that certain immunosuppressive drugs may exert clinically relevant anti-malarial activity. It was therefore designed to assess the intrinsic anti-malarial activity of everolimus, mycophenolic acid, and rapamycin against *Plasmodium falciparum* in an *in vitro* model.

**Methods:**

Three laboratory adapted clones of *P. falciparum* and two isolates were used to assess the potential of mycophenolic acid, rapamycin and everolimus to inhibit *in vitro* growth of *P. falciparum*. The standard histidine rich protein 2 assay was employed and inhibitory drug concentrations (IC) were computed by non-linear regression analysis.

**Results:**

All drugs were associated with complete inhibition of *P. falciparum* growth in *in vitro* assays. Mycophenolic acid demonstrated IC_50_ and IC_90_ values of 5.4 μmol/L and 15.3 μmol/L. Rapamycin inhibited *P. falciparum* growth at 7.2 μmol/L (IC_50_) and 12.5 μmol/L (IC_90_), respectively. Finally, everolimus displayed IC_50_ and IC_90_ values of 6.2 μmol/L and 11.5 μmol/L. There was no difference in *in vitro* activity against chloroquine sensitive or chloroquine resistant parasites.

**Conclusions:**

All immunosuppressive drugs evaluated in this *in vitro* study demonstrated activity against *P. falciparum*. Inhibitory concentrations of mycophenolic acid are within clinically achievable plasma concentrations when used in solid organ transplant recipients. Further *in vivo* evaluation of mycophenolic acid either alone or in combination regimens may prove promising for the concomitant prevention of *P. falciparum* in solid organ transplant recipients living or travelling in malaria endemic regions.

## Background

Despite international efforts to control and ultimately eliminate malaria, *Plasmodium falciparum* remains among the three most deadly infectious pathogens in sub-Saharan Africa causing an estimated 627,000 deaths every year [1]. One of the most formidable challenges in the fight against falciparum malaria is the ongoing emergence and spread of drug resistant isolates leading to reduced efficacy and effectiveness of established anti-malarial therapies [2]. Large-scale *in vitro* screening of drug candidates is performed to discover new compounds with anti-malarial activity [3]. However, only a fraction of those with proven *in vitro* activity are successfully developed to a marketed drug mostly due to concerns with safety and tolerability in preclinical or clinical development. Screening of drugs already used in clinical practice for other indications than malaria is another valuable approach to build-up the anti-malarial armamentarium. Drugs which are used for chronic conditions necessitating lifelong treatment may be of particular interest for their use as anti-malarial prophylactic agents.

The ever growing number of patients requiring immunosuppressive therapy after solid organ transplantation is particularly vulnerable for infectious diseases. Whereas rapamycin – commonly referred to as sirolimus – and cyclosporine A have been shown to exhibit anti-malarial activity *in vitro*, no conclusive data are available for everolimus and mycophenolic acid – two of the most widely used immunosuppressive drugs [4]. An immunosuppressive combination treatment with collateral prophylactic efficacy against malaria would constitute an important benefit for solid organ transplant recipients in malaria endemic regions and travellers to tropical countries. This would be of even higher clinical benefit as immunosuppressive drugs are prone to clinically significant drug interactions and the interaction of anti-malarials and immunosuppressive drugs is currently not well understood. This study was, therefore, designed to assess the *in vitro* activity of mycophenolic acid, everolimus and rapamycin against *P. falciparum* to obtain further information about the potential of currently used immunosuppressives as anti-malarial prophylactic drugs.

## Methods

### Laboratory strains and clones

The study was performed with three *P. falciparum* clones with distinct anti-malarial resistance patterns (Dd2, 3D7 and 7G8) and two laboratory-adapted strains from returning travellers. Clones Dd2 and 7G8 are resistant against chloroquine, whereas 3D7 sensitive to all commonly used anti-malarials [5]. Laboratory adapted strains originated from two returning travellers who acquired *P. falciparum* infections in West Africa. These strains demonstrate high level resistance against chloroquine but are sensitive against mefloquine and artemisinin derivatives. Parasites were kept in continuous malaria culture as described previously [6].

### Test compounds

Chloroquine diphosphate salt (Sigma, C6628) was dissolved in double distilled water. Mycophenolic acid (Fluka, 70018), rapamycin (Sigma, R0395) and everolimus (Fluka, 07741) were dissolved in absolute ethanol. All stock solutions were further diluted in double distilled water to obtain respective target concentrations. 96-well culture plates were coated with ascending concentrations of chloroquine (0.003-3 μmol/L), mycophenolic acid (0.6-39 μmol/L), rapamycin (0.2- 13.7 μmol/L) and everolimus (0.2-13 μmol/L). Test plates were dried and kept at 4°C for a maximum of two weeks before use. All drug assays were performed in duplicate with all *P. falciparum* clones and strains.

### Drug sensitivity assay

Cultured parasites were incubated for 72 hours on pre-coated plates and drug sensitivity assays were performed using an ELISA for quantitative measurement of the *P. falciparum* specific histidine rich protein 2 (HRP-2). ELISA plates were coated with commercial monoclonal antibodies and tests were performed as previously described [7]. An at least four-fold increase in HRP-2 concentrations was set as threshold for further statistical analysis of drug sensitivity assays. Statistical analysis was performed and inhibitory concentrations (IC) were calculated using freely available software [8]. This program was developed to analyse data from malarial drug sensitivity tests such as the HRP-2 assay and produces non-linear regression models to calculate individual inhibitory concentrations (IC). No ethical clearance was required for this *in vitro* study following regulations of the Ethics Committee of the Medical University of Vienna.

## Results

### Immunosuppressives

All drugs were tested in parallel against the three clones and two strains of *P. falciparum* resulting in 20 evaluable drug-concentration/growth-inhibition curves. Growth rates were sufficient in all assays for further analysis. Mycophenolic acid led to complete inhibition of *in vitro* growth of *P. falciparum* at 19.5 μmol/L and 39 μmol/l, respectively. Complete growth inhibition was achieved at highest concentrations of rapamycin (13.7 μmol/L) and at 13 μmol/L of everolimus for all tested clones and isolates. Individual 50 and 90 percent inhibitory concentrations are shown in Table 1.

**Table 1:** **Individual IC**_**50**_ **and IC**_**90**_ **values for respective immunosuppressive drugs are shown for each tested parasite sample and for chloroquine resistant strains**, **respectively**

Individual IC _50_ and IC _90_ values ^1^											Median IC _50_ and IC _90_ values	
	3d7		7G8		Dd2		Strain 1		Strain 2		CQr ^2^ parasites	
	IC _50_	IC _90_	IC _50_	IC _90_	IC _50_	IC _90_	IC _50_	IC _90_	IC _50_	IC _90_	IC _50_	IC _90_
Mycophenolic acid	5.3	15.1	5.4	15.3	5.7	16.7	5.4	14.4	6.4	23.7	5.6 (5.4-5.9)^3^	16 (15.1-18.5)
Rapamycin	8.1	12.5	6.7	11.8	6.6	12.3	7.2	12.7	7.8	12.7	6.9 (6.6-7.4)	12.5 (12.2-12.7)
Everolimus	7.5	11.8	5.1	10.7	6.4	11.5	5.8	11.2	6.2	11.6	6 (5.7-6.3)	11.3 (11.1-11.5)
Chloroquine	0.01	0.03	0.19	1.05	0.22	1.09	0.21	0.65	0.27	0.73	0.21 (0.2-0.23)	0.9 (0.7-1.1)

1 in μmol/L, 2 Chloroquine resistant, 3 interquartile range.

### Evaluation of cross-sensitivity between chloroquine and immunosuppressive drugs

As shown by the individual IC values in Table 1, 3D7 was the only chloroquine sensitive parasite tested in this trial. Median results for chloroquine resistant parasites are presented in comparison and no consistent pattern of alterations in anti-malarial activity of immunosuppressive drugs in chloroquine resistant versus chloroquine sensitive parasite samples was observed.

## Discussion

Mycophenolic acid, rapamycin and everolimus reliably inhibited *in vitro* growth of *P. falciparum* in our study. Inhibition was independent of chloroquine susceptibility and was observed in all tested parasites. Therapeutic target concentrations are in the range of around 5.4-32.8 nmol/L for rapamycin and 3–15.7 nmol/L for everolimus when used as immunosuppressive drugs after solid organ transplantations [9]. As the *in vitro* growth inhibition is at considerably higher concentrations, these drugs are unlikely to exert clinically important anti-malarial activity *in vivo*. On the contrary, inhibitory concentrations for mycophenolic acid were within clinically relevant plasma levels in patients using standard immunosuppressive regimens for solid organ transplantation. Peak plasma levels after oral intake of 1 g mycophenolic acid are around 78 μmol/L [10]. Importantly, mycophenolic acid has a relatively long half-life of around 17 hours leading to sustained high plasma concentrations in currently recommended twice oral daily regimens. Trough levels under steady state conditions are around 10 μmol/L indicating the potential for prolonged anti-malarial *in vitro* activity of mycophenolic acid [11]. With all caveats applicable for extrapolation of *in vitro* data to the *in vivo* situation, these results clearly demonstrate the potential of clinically important activity of mycophenolic acid against *P. falciparum*. Although this drug is unlikely to be used as a targeted treatment for malaria, its clinical usefulness as prophylactic agent in transplant recipients may be further investigated. It may be speculated that the use of mycophenolic acid alone – or in combination with other immunosuppressive drugs – may provide sufficient blood schizontocidal activity to preclude the need for additional chemoprophylaxis.

Interestingly, there is a historic link of anti-malarials and immunosuppressive drugs with the 4-aminoquinolines as the most prominent example. Chloroquine – first developed as anti-malarial drug – has known immuno-modulatory effects and is used on a large scale for the treatment of rheumatic diseases including lupus erythematosus [12]. Similarly, cyclosporine A and FK506 possess antiparasitic activity and have been considered as lead agents for new classes of antimicrobial drugs [13]. Mycophenolic acid may, therefore, become yet another example of an immunosuppressive drug with potentially clinically important anti-malarial activity.
